# Temporal context affects the perceived time of visual events

**DOI:** 10.3758/s13423-019-01682-x

**Published:** 2019-12-05

**Authors:** Ljubica Jovanovic, Pascal Mamassian

**Affiliations:** 1 Laboratoire des Systèmes Perceptifs, Département d’études Cognitives, École Normale Supérieure, PSL University, CNRS, 75005 Paris, France; 2 Neuropsychologie Cognitive, Physiopathologie de la Schizophrénie, Inserm, UR 1114, Strasbourg, France

**Keywords:** Perceived time, Precuing, Temporal context

## Abstract

We investigated whether the moment at which an event is perceived depends on its temporal context. Participants learned a mapping between time and space by watching the hand of a clock rotating a full revolution in a fixed duration. Then the hand was removed, and a target disc was flashed within a fixed-interval duration. Participants were to indicate where the hand would have been at the time of the target. In three separate experiments, we estimated the disruption from a distractor disc that was presented before or after the target disc, with a variable time between them. The target was either revealed at the end of the trial or cued beforehand, and in the latter case, was cued by either color or temporal order. We found an attraction to the presentation time of the distractor when both events were attended equally (target revealed at the end). When the target was cued beforehand, the reported time was under- or overestimated, depending on whether the nature of distractor had to be decoded (precued by color) or not (precued by order). In summary, the perceived time of an event is always affected by other events in temporal proximity, but the nature of this effect depends on how each event is attended.

The perception of time is malleable. For example, the perceived duration between two attended events is affected by irrelevant “distractor” events presented before or after them (Burr, Della Rocca, & Morrone, 2013; Karmarkar & Buonomano, 2007; Nakajima, Ten Hoopen, & Van Der Wilk, 1991). The longer the duration between the distractor and the first event, the stronger the bias to perceive the duration between the two attended events as longer. The attraction toward the duration of the distractor interval is explained by a tendency to regularize the sequence of the three events constituting the intervals (Burr et al., 2013; Remijn et al., 1999; Sawai, Sato, & Aihara, 2012). Although it is implicit in the regularization hypothesis, we do not know whether the perceived time of a single event is affected by its context. Here, we asked whether the moment *when* an event is perceived is affected by other events presented in its temporal proximity.

As we investigate how the perceived time of an event is influenced by its context, it will be informative to recall how the perceived spatial position of visual events is also affected by other events in their spatial and/or temporal proximity. For example, the perceived spatial position of a target can be either attracted to or repelled from a distractor, depending on the temporal order of the two (Chien, Ono, & Watanabe, 2011; Chow, Gozli, & Pratt, 2014; Ono & Watanabe, 2011; Suzuki & Cavanagh, 1997). In the multiple-object spatial-tracking task, perceived space can be either compressed or expanded, depending on whether or not the events are attended to (Liverence & Scholl, 2011). We aimed to extend these findings to the moment when an event is perceived. In three experiments, we varied when and how the target was cued. The target was revealed only at the end of the trial (“postcued by color”) or was cued beforehand by either its color (“precued by color”) or temporal order (“precued by order”).

## Method

### Stimuli

The stimuli were red and green discs of radius 1 deg of visual angle (dva), flashed briefly (33 ms). The fixation point was a 0.5-dva white disc that changed its luminance to dark gray as a preparation signal, just before the beginning of the trial. At the beginning of the trial, the fixation location changed into a placeholder for the stimulus, a white circle, that had the same size as the stimuli. During the experiment, a white circle representing the face of a clock was always present. The hand of the clock was shown only during the familiarization phase. The face and the hand of the clock had a radius of 2.5 dva and the same white color. Each trial started and ended with a 33-ms pure tone of frequency 1 kHz.

### Apparatus

The experiments were conducted in a dimly lit room and were created using Matlab R 2016a and Psychtoolbox 3 (Brainard, 1997) running on a MAC Pro Quadro-Core Intel Xeon with OSX 10.5.8. The stimuli were presented on an LCD flat screen (ViewSonic V3F245) with diagonal 24 in., resolution 1,920 × 1,080 pixels, and a refresh rate of 60 Hz. The viewing distance was 50 cm.

Analysis of the data was conducted in the R Studio environment, using the lme4 package (Bates, Mächler, Bolker, and Walker, 2015) for mixed-effect regression analysis. We excluded trials with errors greater than 120 deg from the analyses (less than 5% of the trials were excluded).

### Participants

In all, 24 participants took part in the experiments (including 18 females and six males; overall mean age = 24.6 years). All but one participant (the first author, who participated in the “postcued by color” experiment) were naive to the purpose of the study and gave written informed consent. We tested eight participants in each experiment, a sample size chosen on the basis of similar previous studies (e.g., Libet, Gleason, Wright, & Pearl, 1983; Liverence & Scholl, 2011; Suzuki & Cavanagh, 1997). The experiment was conducted in agreement with the Declaration of Helsinki and local ethics regulations.

### Procedure

**Fig. 1:**
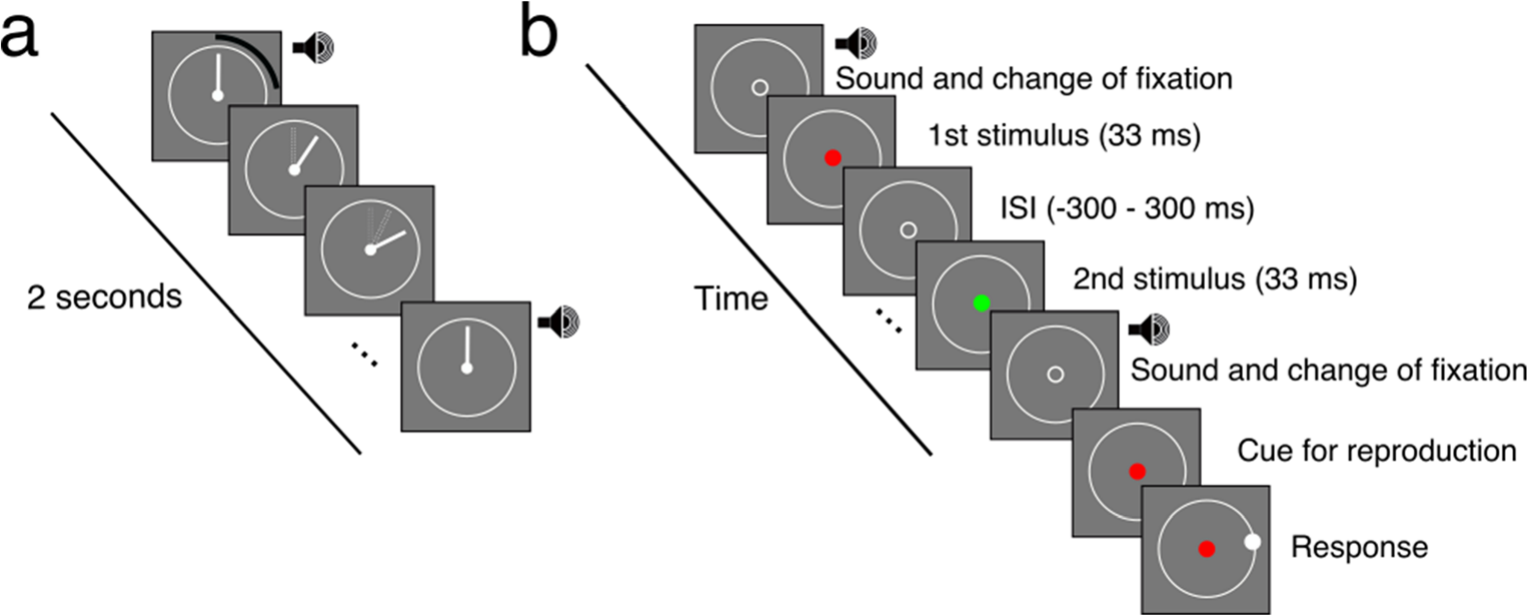
Chronology of the familiarization phase (a) and of an experimental trial (b). (a) Familiarization with trial duration. Participants were presented with a clock at the beginning of the experiment and after each break. The hand of the clock rotated at a constant velocity over 2 s for one full revolution. A brief 1-kHz pure tone was presented at the beginning and end of each revolution, as an additional cue to facilitate learning of the trial duration. (b) Illustration of the chronology of the “postcued by color” experiment. During the experiment, the hand of the clock was removed, but the white circle representing the face of the clock remained on the screen. At the start of the trial, the fixation disc changed to a placeholder for the stimulus, and a brief tone was presented. After a random delay, two stimuli were presented successively, one red and one green, in random order. The trial lasted 2 s, and the end of the trial was marked by a change of the placeholder to a fixation disc and a brief tone. When the trial ended, one of the two discs was presented again, to indicate the color of the target disc. Participants moved the mouse cursor to place it on the clock face at the location where the hand of the clock would have been at the time of the target presentation. The procedure was similar in the “precued” experiments, except that participants knew beforehand the color (red/green) or the temporal order (first/second) of the target and the distractor. In the “precued” experiments, the two colors and temporal orders were tested in separate blocks.

At the beginning of the experiment, participants were familiarized with a fixed trial duration, by watching the hand of a clock rotating at a constant speed, one cycle in 2 s. To provide additional cues and facilitate learning of the trial duration, a brief tone (33 ms, 1 kHz) was presented at the beginning and the end of each revolution (Fig. 1a). In the remainder of the experiment, the hand was not presented. The clock face was represented as a circle during the trial, and the two tones were presented at the beginning and end of each trial. Participants were asked to fixate the fixation circle presented at the center of the clock face. After a variable duration from the beginning of the trial, two stimuli were briefly presented in temporal sequence. Participants were asked to attend to the time from the beginning of the trial and to estimate when the stimuli were being presented, relative to the beginning and end of the trial. Participants gave their responses at the end of the trial by placing a cursor on the face of the clock at the location where the hand of the clock would have been at the time of one of the stimuli (Fig. 1b). Participants always reported when one of the two stimuli was presented. We will refer to the stimulus they were asked to report as the *target*, and the other stimulus of the pair as the *distractor*. On each trial, the presentation time of the target within the trial interval was chosen randomly. The time of the distractor relative to the target was chosen following the method of constant stimuli and could take one of ten levels, from – 300 ms (the distractor before the target) to + 300 ms (the distractor after the target). In the “precued by color” and “postcued by color” experiments, we also presented trials in which only the target was presented (9% of trials). We never presented a stimulus (target or distractor) within 150 ms after the beginning or before the end of the trial.

In three experiments, we varied how and when the target was revealed. In the “postcued by color” experiment, participants were asked to attend to both events, and only at the end of the trial was the target revealed by its color. In the other two experiments, participants knew in advance of each trial which event they would be asked to report, and in separate blocks of trials, the target was cued either by color (“precued by color”) or temporal order (“precued by order”). In the “postcued by color” and “precued by order” experiments, the two colors were randomly assigned to the two stimuli on each trial.

## Results

### When an event is perceived is affected by other events presented before or after it

**Fig. 2:**
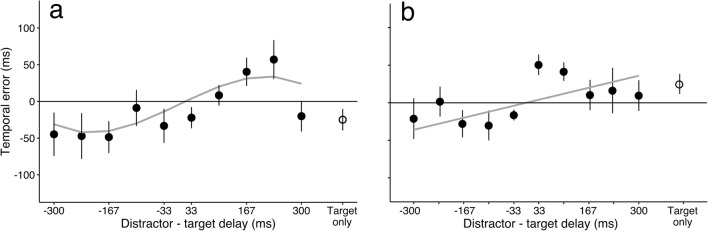
Temporal errors in the “postcued by color” experiment. Temporal errors in reporting the perceived time of the target, whose identity was revealed at the end of the trial, are shown for the main experiment, in which the trial duration was 2 s (a), and for the control experiment, in which the trial duration was 1.5 s (b). In both panels, the average temporal error across participants is plotted against the different temporal delays between distractor and target (positive delays indicate that the target was presented before the distractor). The reported time of the target was biased toward the moment when the distractor was presented. The target was reported later (positive temporal error) if the distractor was presented after, and earlier if the distractor was presented before. The gray lines correspond to the best polynomial fits to the data. Error bars indicate standard errors of the means.

To estimate the accuracy of the perceived time of the target, we calculated the temporal error as the difference between the reported and actual presentation times. Figure 2 shows the results of the “postcued by color” experiment, in which the target was identified at the end of the trial by revealing its color. The average error across participants is shown against the different temporal delays between distractor and target.

In this “postcued by color” experiment, the reported target time was attracted to the presentation time of the distractor (Fig. 2a). The moment when the target was perceived was reported as being later if the distractor was presented after the target. We quantified the effect by means of a linear mixed-effect model. The temporal error for subject *s* and each condition *i* of the delay between target and distractor was modeled as a third-order polynomial:

$$ {E}_{is}=\left({b}_0+{s}_{0s}\right)+{b}_1{D}_i+{b}_2{D_i}^2+{b}_3{D_i}^3+{n}_{is}, $$

where

$$ {D}_i=\mathrm{Distractor}\_\mathrm{Time}(i)\hbox{--} \mathrm{Target}\_\mathrm{Time}(i), $$

$$ {E}_{is}=\mathrm{Reported}\_\mathrm{Time}\left(i,s\right)\hbox{--} \mathrm{Target}\_\mathrm{Time}(i), $$

and *b*_0_, *b*_1_, *b*_2_, and *b*_3_ are regression coefficients for the polynomial terms. Variability at the subject level was modeled by a random intercept parameter *s*_0*s*_ that represented deviations from intercept *b*_o_, and *n*_*is*_ was the residual error term for each subject.

We observed a significant effect of the delay between the target and the distractor; estimates of when the stimulus was presented were biased toward the timing of the distractor (*b*_1_ = 0.264, *SE* = 0.065, *t* = 3.998, *p* < .01). Also, the cubic term of the polynomial was significant (*b*_3_ = – 1.972, *SE* = 0.936, *t* = – 2.106, *p* < .05), indicating that the effect of the distractor decreased with increased temporal distance between the target and the distractor (when the distractor was sufficiently remote from the target, its attraction vanished). Excluding the cubic term significantly decreased the goodness of fit of the model, as we assessed by a comparison of the models’ log-likelihoods with a chi-square test [*χ*^2^(1) = 4.413, *p* < .05].

To confirm that our results can be generalized to trials that have durations other than 2 s, we conducted an additional experiment. The procedure, stimuli, and distractor conditions were the same as in the “postcued by color” experiment, and the only difference was that the duration of the trial was now fixed to 1.5 s. We recruited eight new participants for this experiment. The average temporal error across participants is shown in Fig. 2b. We again observed a significant positive effect of the distractor (*b*_1_ = 0.116, *SE* = 0.048, *t* = 2.411, *p* < .05). The second- and third-order terms, however, were not significant in this replication (*b*_2_ = – 0.0935, *SE* = 0.112, *t* = – 0.835, *p* = *.*404, and *b*_3_ = – 0.911, *SE* = 0.688, *t* = – 1.324, *p* = *.*186, respectively). Importantly, the main effect of the presentation time of the distractor on the perceived time of the target was significant in both experiments, and in both cases the target was attracted to the distractor (*b*_1_ > 0).

**Fig. 3:**
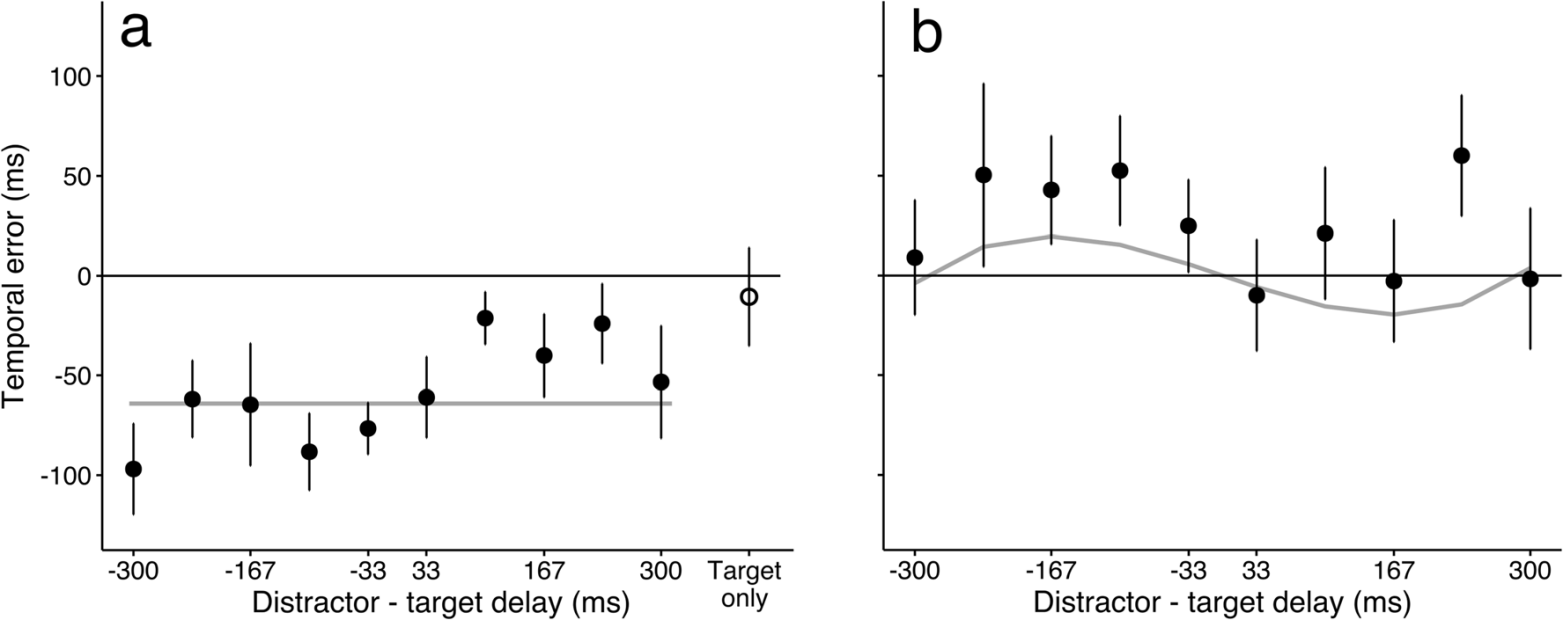
Temporal errors in the two “precued” experiments. (a) Bias to report events earlier in the “precued by color” experiment. The average temporal error across participants is plotted against the different temporal delays between the distractor and target. There was a large bias to report targets earlier when the color of the target was known beforehand. The gray line corresponds to the bias, a significant intercept from the statistical model. (b) Repulsion away from the timing of the distractor in the “precued by order” experiment. The reported time of the target was biased away from the moment when the distractor was presented, especially in the condition in which the distractor was presented before the target (the target was reported as appearing later). The gray line corresponds to the polynomial fit to the data. In both panels, error bars indicate standard errors of the means.

We conducted the same analysis for the other two experiments. In the “precued by color” experiment, the target was revealed by its color before the trial started. In this experiment, we found an overall large negative bias for the reported time of the target (Fig. 3a). This bias was revealed by a significant intercept (*b*_0_ = – 0.064, *SE* = 0.019, *t* = – 3.307, *p* < .01) in the polynomial fit. None of the higher-order terms were significant (*b*_1_ = 0.110, *SE* = 0.064, *t* = 1.706; *b*_2_ = 0.154, *SE* = 0.148, *t* = 1.040; *b*_3_ = – 0.736, *SE* = 0.917, *t* = – 0.824).

In the “precued by order” experiment, the target was also revealed before the trial started, but this time it was the stimulus order that mattered (first or second). In this experiment, we again found an effect of the distractor, but this time the perceived time of the target was biased away from the distractor (Fig. 3b). This bias was revealed by a significant negative relationship between the reported time of the target and the presentation time of the distractor (*b*_1_ = – 0.176, *SE* = 0.06, *t* = – 2.911, *p* < .01). As in the “postcued by color” experiment, the cubic term of the polynomial was significant (*b*_3_ = 2.093, *SE* = 0.856, *t* = 2.444, *p* < .05), indicating that the effect of the distractor decreased with increasing temporal delay between the target and the distractor. Excluding the cubic term significantly decreased the goodness of fit [*χ*^2^(1) = 5.9731, *p* < .05].

## Discussion

We found that the moment when a target is perceived is biased by the presentation of other events, and that different cueing methods create different biases. In the “postcued by color” experiment, the target on average was reported later (positive temporal error) if the distractor was presented after it, and earlier (negative temporal error) if the distractor was presented first. When the target was precued by color, targets were reported earlier than they were presented. Finally, in the “precued by order” experiment, the target was reported later if the distractor was presented before it. What could be the reason for these different effects of the temporal context on the perceived time of a target?

The “postcued by color” experiment forced participants to attend equally to both the target and a distractor, because the target was only revealed at the end of the trial. In this case, the reported time of the target was attracted toward the presented time of the distractor. We chose the duration of the trial to be either 2 s or 1.5 s as a trade-off. These durations needed to be short enough so that the uncertainty of temporal perception was relatively small (the longer the duration, the more uncertain the estimate; Gibbon, 1977), and long enough so that two visual events could be presented. Importantly, for both trial durations, we found a consistent bias of the perceived time toward the average time of the two events. This finding is broadly in agreement with previous work revealing how temporal context can bias the perceived duration of events (Burr et al., 2013; Jazayeri & Shadlen, 2010; Nakajima et al., 1991; Sawai et al., 2012). Critically, though, we showed here that the temporal proximity of two objects can bias the perceived time of a single brief event.

Attending to one stimulus of the pair revealed a different effect of the distractor on the reported time of the target. Interestingly, the reported time depended on what was being attended (Zakay, 1998). When participants knew the color of the target to be presented, it was reported earlier than it was actually presented. Although this bias is reminiscent of the prior-entry effect (in which attended events are perceived earlier than not-attended ones; Titchener, 1908), it was observed only when two events were presented in a trial. When only the target was presented, there was no bias, even though the color of the event was still the color attended in that block, suggesting that the bias is specific to conditions in which two stimuli are encoded in rapid succession.

It is known that humans sometimes fail to properly monitor the duration of certain perceptual processes or motor actions. For example, the perceived duration of saccades or attentional shifts is inaccurate (Jonikaitis, Deubel, & de’Sperati, 2009; Morrone, Ross, & Burr, 2005), although the duration can be compensated for later (Yarrow, Haggard, Heal, Brown, & Rothwell, 2001; Yarrow, Whiteley, Haggard, & Rothwell, 2006). Similarly, the durations of blinks are ignored (Riggs, Volkmann, & Moore, 1981). Our findings in the “precued by color” experiment could be explained by an underestimation of the time needed to process the color of the distractor or make an inference about which of the two stimuli is the target. Importantly, no bias was observed when only one stimulus was presented in the “precued by color” experiment, confirming that a distractor is necessary for the effect.

When an event was known to be a distractor even before it was processed (“precued by order” condition), the moment when the target was perceived was repelled from the time of the distractor. At this stage, we can only speculate on the reasons why attending to color and to temporal order creates different biases. Unlike attending to the color of the target, cueing with the temporal order of the target does not require processing of the distractor stimulus. Nevertheless, the distractor cannot be ignored, and it is possible that the time needed to process it is overestimated, so that the target is reported later.

The overall aim of the study was to investigate whether the moment when an event is perceived is affected by other events presented before or after it. We varied the duration between the target and the distractor in ten different steps. The largest temporal interval between the two events was 300 ms, so the two events were predictive of each other (when one event was detected, the other event should happen within the next 300 ms). It is possible that this experimental design enhanced any interaction between the two events. A design in which the distractor is presented at a completely random time relative to the target would reduce this possible confound. However, since such a study would require a very large number of trials in order to obtain reliable estimates for the different target–distractor temporal relationships, we decided to restrict the design to a relatively modest target–distractor interval range. That said, temporal predictability should affect temporal estimation mostly in the condition in which the target was presented after the distractor, and in contrast to that prediction, we found symmetric effects before and after target presentation.

In summary, the perceived time of a single brief event can easily be biased to appear earlier or later than when it was presented, simply by presenting another object in near temporal proximity. Importantly, the perceived time is determined by the manner in which we attend to the other, distracting events.
